# Cold Atmospheric Plasma Selectively Targets Neuroblastoma: Mechanistic Insights and In Vivo Validation

**DOI:** 10.3390/cancers17213432

**Published:** 2025-10-25

**Authors:** Ligi Milesh, Bindu Nair, Ha M. Nguyen, Taylor Aiken, J. Leon Shohet, Hau D. Le

**Affiliations:** 1 Division of Pediatric Surgery, Department of Surgery, University of Wisconsin-Madison, Madison, WI 53705, USA; milesh@wisc.edu (L.M.); bindu.nair@bpt.eurofinsus.com (B.N.); hn4gq@missouri.edu (H.M.N.); aikent1@mskcc.org (T.A.); 2 Department of Biomedical Engineering, University of Wisconsin-Madison, Madison, WI 53705, USA; 3 Department of Electrical and Computer Engineering, University of Wisconsin-Madison, Madison, WI 53705, USA; shohetjl@gmail.com; 4 Carbone Cancer Center, University of Wisconsin-Madison, Madison, WI 53705, USA

**Keywords:** Cold atmospheric plasma (CAP), neuroblastoma (NB), viability, reactive oxygen species (ROS), lipid peroxidation, DNA damage, apoptosis, cell viability, in vitro, in vivo, tumor models, novel therapy

## Abstract

**Simple Summary:**

Neuroblastoma is one of the most challenging pediatric cancers to treat, especially in high-risk patients where recurrence is common. Cold Atmospheric Plasma (CAP) is a novel approach that may kill cancer cells with less damage to normal cells. In this study, we investigated the use of CAP in neuroblastoma cells using both in vitro and in vivo models. CAP selectively induced more cancer cell death than normal cell death by generating reactive oxygen species, damaging DNA, and promoting apoptosis. It also delayed tumor growth and regrowth in vivo. These findings support CAP as an additional promising modality for current neuroblastoma treatment strategies, particularly post-surgery. Future experiments will use ROS scavengers (e.g., N-acetylcysteine) and pro-oxidant challenges to confirm the causal role of reactive oxygen species in CAP-induced apoptosis and to further define dose ranges that maximize tumor selectivity while minimizing normal tissue toxicity.

**Abstract:**

Background: Neuroblastoma (NB) presents significant challenges in pediatric oncology, particularly in high-risk cases where local recurrence occurs in ~35% of patients. Cold Atmospheric Plasma (CAP) has emerged as a promising treatment due to its selective cytotoxicity toward cancer cells while sparing normal cells. Methods: This study assessed CAP efficacy using in vitro NB cell lines (SK-N-AS and LAN-5) and in vivo xenograft murine models. In vitro, CAP was applied via a helium jet, and cellular responses were evaluated for viability, reactive oxygen species (ROS), lipid peroxidation, DNA damage, and cell cycle, while apoptosis was measured by Annexin V/PI flow cytometry. In vivo, CAP was applied to unresected tumors and residual tumors after incomplete resection. Tumor regrowth was monitored, and histological analysis was performed. Results: CAP reduced NB cell viability in a dose- and time-dependent manner by increasing intracellular ROS and lipid peroxidation. CAP-treated NB cells showed a 50% rise in oxidative DNA damage, a two-fold increase in apoptosis, and alterations in cell-cycle progression, while normal fibroblasts showed modest effects. CAP predominantly induced apoptosis, though secondary necrosis appeared with prolonged exposures, consistent with caspase-3 and PARP pathways. In xenografts, CAP reduced tumor diameter by 60% and increased caspase-3-positive cells, with minimal effects on normal tissue. Conclusions: CAP demonstrates strong therapeutic potential as a targeted, non-invasive NB treatment, particularly for residual tumors near vascular structures with consistent exposure times (60–300 s).

## 1. Introduction

Neuroblastoma (NB) is the most common extracranial solid tumor of childhood, accounting for over 7% of malignancies in children under 15 years old and approximately 15% of pediatric cancer-related deaths globally [1]. It arises from neural crest cells of the sympathetic nervous system, and predominantly affects infants and young children, with about 90% of cases diagnosed before the age of five [2]. The clinical spectrum of NB ranges widely, from localized tumors that may regress spontaneously to high-risk cases characterized by widespread metastasis and poor treatment outcomes [3].

Advanced NB presents significant therapeutic challenges due to its aggressive nature, early metastasis to bones, bone marrow, liver, and lymph nodes, and the difficulty in achieving complete surgical resection [4]. Despite advances in multimodal therapies such as surgery, chemotherapy, and radiotherapy, the prognosis for high-risk NB remains poor, with less than 40% survival rates beyond five years [5]. This is exacerbated by the tumor’s propensity to develop resistance to conventional treatments, necessitating innovative therapeutic approaches [6].

Surgical resection is one of the primary treatment modalities for high-risk NB, but is challenged by the tumor’s infiltrative nature, frequently leaving residual cancer cells that require adjunctive therapies [7]. These therapies, however, carry risks including secondary cancers, organ damage, and developmental delays, particularly concerning young patients [8]. Despite multi-modality treatment, local recurrence occurs in up to 35% of patients [9].

Cold atmospheric plasma (CAP) has emerged as a promising avenue in cancer therapy. CAP, generated at room temperature and atmospheric pressure, comprises ions, electrons, photons, and reactive oxygen and nitrogen species (RONS), which collectively exert cytotoxic effects on cancer cells while sparing normal tissues [10,11]. CAP’s ability to induce apoptosis, disrupt cellular functions, and modulate the tumor microenvironment makes it a compelling candidate for targeted cancer therapy [12].

Recent studies have demonstrated the efficacy of CAP treatment in various cancers, including melanoma, glioma, and neuroblastoma, showing reductions in tumor size and improved survival rates in preclinical models [13,14,15]. The mechanism of CAP on these tumors has been proposed to occur via cell cycle arrest and apoptosis induction in cancer cells, mediated by RONS, particularly hydroxyl radicals (•OH) [16]. A distinct pre-G1 peak was not detected, consistent with NB, where apoptosis is more reliably assessed by Annexin V/PI rather than pre-G1 fractions.

In addition, although pioneering, previous work examined CAP in a murine NB model and was limited to a single murine cell line without a mechanistic analysis [13]. Our study expands this by using two human NB lines, normal fibroblasts, mechanistic assays, and in vivo xenografts, thereby establishing novelty and translational relevance.

This study aims to investigate CAP as a potential therapeutic strategy, specifically for NB. We sought to understand how CAP can selectively induce apoptosis in NB cells using in vitro experiments. We then aimed to investigate the efficacy of CAP as primary therapy or as an adjunct to surgery to decrease residual disease, thereby reducing the local recurrence rate.

## 2. Materials and Methods

### 2.1. CAP Device

**Figure 1:**
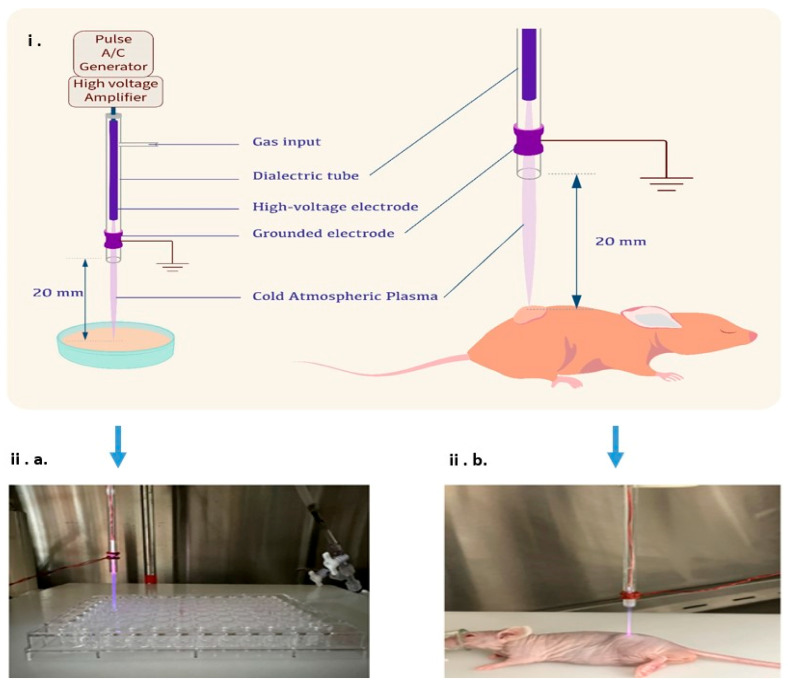
CAP jet device developed at UW–Madison and its application. (**i**) Schematic representation of the CAP system constructed using the dielectric barrier discharge (DBD) method. The setup includes a pulse A/C generator connected to a high-voltage amplifier that produces voltages between ±10 kV at 20 kHz. Helium is used as the carrier gas, and the plasma jet is directed toward cultured cells or tumors on an animal, with an adjustable nozzle distance ranging 1–10 cm. (**ii**) Representative application of CAP in experimental models. (**ii. a**) The left panel illustrates CAP exposure to neuroblastoma cells in a 96-well plate (in vitro), and (**ii. b**) the right panel shows CAP application on a subcutaneous tumor in a nude mouse (in vivo). Exposure times ranged from 60 to 300 s, depending on assay type.

The helium CAP jet device based on the dielectric barrier discharge (DBD) method (Figure 1) was custom-built by our team at the University of Wisconsin–Madison and optimized for both in vitro and in vivo applications. The system operates as a jet-type CAP generator, utilizing helium as the carrier gas, with a flow rate adjustable between 0 and 3 standard liters per minute (slm). High-voltage pulses ranging from −10 kV to +10 kV at a frequency of up to 20 kHz are applied, enabling precise modulation of plasma intensity. The nozzle-to-target distance can be adjusted from 10 mm to 100 mm, offering flexibility to tailor treatment conditions for different experimental setups.

### 2.2. Experimental Design

**Figure 2:**
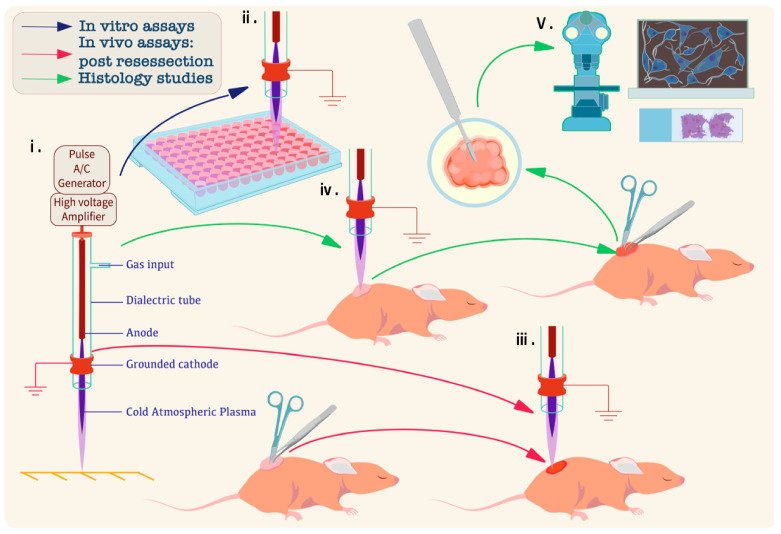
Schematic overview of CAP treatment workflow in NB models. (**i**) CAP Jet System: Schematic of the CAP device showing the DBD configuration comprising a dielectric tube, gas input (helium), anode, grounded cathode, and CAP generated via high-voltage pulses from a pulse A/C generator and amplifier. (**ii**) In Vitro Assays: NB cells cultured in 96-well plates are treated with CAP to assess effects on cell viability, proliferation, and molecular responses. (**iii**) In Vivo Resection Model: CAP is applied to the tumor bed immediately following incomplete surgical resection to target residual cancer. (**iv**) In Vivo Non-Resection Model: CAP is administered directly to the unresected tumor, simulating a non-resection or primary treatment approach. (**v**) Histological and Microscopy Analysis: Tumors excised post-treatment are processed for histological staining and microscopic evaluation to assess CAP-induced molecular and morphological alterations.

A schematic overview of the study design is presented in Figure 2. This illustration provides a visual summary of the experiment’s sequential workflow for investigating CAP on NB, including both in vitro and in vivo models, as well as subsequent histological and microscopy-based analyses.

### 2.3. Cell Culture

NB cell lines (SK-N-AS and LAN-5) provided by Dr. Mario Otto’s lab (University of Wisconsin-Madison) and normal human dermal fibroblasts (hDF) [PCS-201-010], obtained from the American Type Culture Collection (ATCC, Manassas, VA, USA), were utilized for the study. Cells were cultured in Dulbecco’s Modified Eagle’s Medium (DMEM) (Catalog No. 11965092, Gibco, Thermo Fisher Scientific, Waltham, MA, USA) or RPMI 1640 (Catalog No. 11875093, Gibco, Thermo Fisher Scientific, Waltham, MA, USA) supplemented with 10% fetal bovine serum (FBS (Cat. No. 26140-079, Gibco, Thermo Fisher Scientific, Waltham, MA, USA), 50 units/mL penicillin, and 100 µg/mL streptomycin (Catalog No. 15140122, Life Technologies, Carlsbad, CA, USA) and were incubated in a 5% CO_2_ environment at 37 °C in an incubator, following standard methods. Cells were passaged when confluency reached 70–90%, and all experiments were conducted after 2–3 passages. S.K-N-AS is MYCN non-amplified with TP53 mutation; LAN-5 is MYCN-amplified and ALK wild-type. This helps interpret differential CAP sensitivity.

### 2.4. Cell Viability Assay

Cells were counted, and approximately 10,000 cells were seeded in a 96-well plate (Corning Inc., Corning, NY, USA). After 24 h of incubation, treated cells were exposed to CAP for 60, 120, and 180 s. Control cells were not treated with anything. Helium-treated cells were exposed to helium gas for 60, 120, and 180 s as an additional control. Following these treatments, cells were incubated at 37 °C for 24, 48, or 72 h. Cell viability was measured using Cell Counting Kit-8 (CCK-8) (Catalog No. CK04, Dojindo Molecular Technologies, Rockville, MD, USA). Specifically, 10 µL of CCK-8 reagent was added to each well and incubated for one hour. The optical density (OD) at 450 nm was measured using a microplate reader (Molecular Devices, San Jose, CA, USA). The percentage of viable cells post-treatment relative to the untreated control was presented as cell viability.

### 2.5. Quantification of Intracellular ROS Levels Using H2DCFDA and Highly Reactive ROS (hROS) Using HPF Probe [hROS = Highly Reactive Oxygen Species; HPF = Hydroxyphenyl Fluorescein]

Intracellular ROS formation in cells after CAP treatment was estimated using the fluorescent dye dichlorofluorescein diacetate (H2DCFDA) (Catalog No. D399, Invitrogen, Thermo Fisher Scientific, Waltham, MA, USA). H2DCFDA is a nonpolar compound that is converted into a polar derivative (dichlorofluorescein) by cellular esterase after incorporation into the cells. On the day of the experiment, the growth medium was replaced with Hank’s Balanced Salt Solution (HBSS) (Catalog No. 14175095, Gibco, Thermo Fisher Scientific, Waltham, MA, USA) containing 10 μM H2-DCF-DA dye. CAP-treated and helium-treated (60, 120, or 180 s) or untreated cells were incubated for one hour. After washing with pre-warmed PBS, intracellular fluorescence was measured using an excitation of 485 nm and an emission of 530 nm with a microplate fluorescence reader (Molecular Devices, CA, USA). A novel fluorescence probe 2-[6-(4′-hydroxy)phenoxy-3H-xanthen-3-on-9-yl] benzoic acid (HPF; Catalog No. 10159, Cayman Chemical, Ann Arbor, MI, USA) was used at a final concentration of 10 μM to detect hROS, such as hydroxyl radical and peroxynitrite formation, in the cells. The process was similar to what was mentioned previously. HPF exhibits a bright green fluorescence upon reaction with hydroxyl radical (excitation/emission maxima ∼490/515 nm).

### 2.6. Measurement of Lipid Peroxidation Using C11-BODIPY 581/591

C11-BODIPY 581/591 (Catalog No. D3861, Invitrogen, Thermo Fisher Scientific, Waltham, MA, USA), a fluorescent probe, was used to measure lipid peroxidation and antioxidant efficacy in living cells. hDF and NB cells were cultured in their respective media. C11-BODIPY 581/591 was added to the cells post-CAP treatment or Helium-treatment for 60, 120, and 180 s, alongside untreated controls, at a final concentration of 10 µM. After an hour of incubation, fluorescence intensity was measured using the microplate fluorescence reader (Molecular Devices, San Jose, CA, USA).

### 2.7. Lipid Hydroperoxide Assay

Lipid peroxidation, a well-established mechanism of cellular injury and an indicator of oxidative stress in cells and tissues, was assessed using the Lipid Peroxidation (LPO) assay kit (Catalog No. 10009055, Cayman Chemical, Ann Arbor, MI, USA). A quantitative extraction method, following the kit’s protocol, was employed to extract lipid hydroperoxides into chloroform, which was used directly for the assay. Absorbance was read at 500 nm using the multimode plate reader (Molecular Devices, San Jose, CA, USA). A dose–response curve of absorbance units versus concentration in nmol was calculated.

### 2.8. 8-Hydroxy-2′-deoxyguanosine (8-OHdG) Quantitation for Oxidative DNA Damage

Oxidant-induced DNA damage, indicated by 8-OHdG, was quantified using the OxiSelect™ Oxidative DNA damage ELISA kit (Catalog No. STA-320, Cell BioLabs, San Diego, CA, USA), following the manufacturer’s instructions. After 48 h of CAP treatment lasting 60 s, genomic DNA was isolated from cells using a DNA extraction kit (Catalog No. K0721, Thermo Scientific, Waltham, MA, USA) and converted into single-stranded DNA. The control group received no CAP treatment. DNA samples were digested with nuclease P1 (Catalog No. M0660S, New England Biolabs, Ipswich, MA, USA), followed by incubation with 10 units of alkaline phosphatase (Catalog No. M0371S, New England Biolabs, Ipswich, MA, USA) for one hr at 37 °C in 100 mM Tris, pH 7.5. A 50 μL mixture of 8-OHdG standard or sample was added to the well of the 8-OHdG conjugate-coated plate and incubated for 10 min at room temperature (RT). Following this, 50 μL diluted anti-8-OHdG antibody was added to each well and incubated for one hour at RT. After several washes, a 100 μL mixture of diluted secondary antibody-enzyme conjugates was added to all wells, which were then incubated for one hr at RT. After three washes, 100 μL of substrate solution was added, and the mixture was incubated for 5 min. The reaction was stopped with 100 μL of the stop solution, and the absorbance was measured at 450 nm using the multimode plate reader. Results were standardized to the total DNA concentration.

### 2.9. Apoptosis Assay Using Flow Cytometry

Cells were treated with CAP or Helium for 60, 120, or 180 s. Control cells were not treated. Apoptosis was detected using a FITC Annexin V/Dead Cell Apoptosis Kit (Catalog No. 556547, BD Pharmingen, San Diego, CA, USA). Trypsinized cells were washed twice with PBS, pelleted, and resuspended in the binding buffer. They were then incubated with 5 µL of FITC Annexin V and 5 µL of 100 µg/mL propidium iodide (PI) for 15 min at room temperature in the dark. After 15 min incubation, 400 µL of binding buffer was added to make a 500 µL final solution. Flow cytometry was performed within an hour using an Attune NxT Flow Cytometer (Thermo Fisher Scientific, Waltham, MA, USA), and results were analyzed using FlowJo 10 software (FlowJo LLC, Ashland, OR, USA).

### 2.10. Cell Cycle Analysis

A total of 10,000 hDF and SK-N-AS cells were seeded into each of the 96 wells. The cells were allowed to grow and proliferate for 24 h. The cells were then treated with CAP for 60, 180, and 300 s and incubated for 24 h. Control cells received no CAP treatment. After incubations, the cells were trypsinized and prepared for cell cycle analysis. At 300 s, SK-N-AS cells showed partial redistribution, likely due to checkpoint adaptation, contrasting with a stronger arrest at 180 s.

#### Sample Preparation for Cell Cycle Analysis

Cells from each treatment group were collected and pooled to achieve a density of approximately 50,000 cells per tube. These samples were stained with a (Propidium Iodide (PI) (nuclear dye) at a final concentration of 50 µg/mL) using a cell cycle analysis kit (Catalog No. ab287852, Abcam, Cambridge, UK) according to the manufacturer’s standard protocol and incubated overnight at 4 °C in the dark. Following incubation, cell cycle distribution was analyzed using flow cytometry. The results were processed using FlowJo 10 software (FlowJo LLC) to evaluate CAP-induced changes in cell cycle dynamics.

### 2.11. CAP Treatment on Co-Culture

For co-culture experiments, 10,000 cells/well Green Fluorescent Protein (GFP)-labeled neuroblastoma cells were grown on 10,000 cells/well Red Fluorescent Protein (RFP)-labeled fibroblasts for 24 h. The cells were treated with CAP or Helium for 120, 180, or 240 s. Later, the GFP-positive and RFP-positive cells were sorted. The viability of both cell types was confirmed to be more than 90% before treatment. Cells were treated with CAP, and images were captured under a fluorescent microscope (Leica Microsystems, Wetzlar, Germany).

### 2.12. In Vivo CAP Treatment

Four-week-old female and male nude mice were obtained from The Jackson Laboratory (Bar Harbor, ME, USA) for the in vivo experiments (Figure 1). These mice were housed in the containment zone of the animal laboratory at the Research Animal Resource Center (RARC) at UW-Madison. Throughout the study, all mice were handled and cared for in strict accordance with the standard guidelines prescribed by the Institutional Animal Care and Use Committee (IACUC) protocol.

#### 2.12.1. Incomplete Resection Model

A total of 8 nude mice were utilized in the present study. Subcutaneous injections of SK-N-AS cells tagged with GFP/LUC+ were administered into the right or left dorsal side of each mouse. Tumor growth was monitored every 2 days by measuring the largest dimension using a caliper. Once the tumor’s largest dimension reached 1–3 mm, resection surgery was performed, aiming to remove approximately 95% of the tumor tissue. For the treated group, CAP treatment was applied for 300 s to the residual tumor. In contrast, the control group had surgery but received no CAP treatment. Following the surgery, the outer skin was closed with staples, and triple antibiotics were applied to the surgical site to prevent infection. The mice were then monitored post-surgery to observe tumor regrowth and document their progress for further analysis. Towards the end of the experiment (63 days), or if the tumor diameter reached a maximum size of 20 mm or more, or in cases where mice appeared moribund, the mice were sacrificed.

#### 2.12.2. Non-Resection Model

A total of 13 nude mice were included in this experiment. Subcutaneous injections of SK-N-AS cells tagged with GFP/LUC+ were administered into the right or left dorsal side of each animal. Tumor growth was measured by the largest dimension every 2 days. Once the tumor’s largest dimension reached 1–3 mm, the mice received CAP treatment applied directly onto the tumor for 300 s, while the control mice received no treatment. Both groups were continuously monitored for tumor growth and documented for further analysis. Towards the end of the experiment (72 days), or when the tumor diameter reached a maximum diameter of 20 mm or more, or in cases where mice appeared moribund, mice were sacrificed, and the data was censored.

#### 2.12.3. Tumor Diameter Analysis

Tumor regrowth and growth data obtained from both the resection and non-resection studies were processed using Microsoft Excel (Microsoft Office 365, Microsoft Corp., Redmond, WA, USA), and the resulting data were used to generate graphs. To assess survival outcomes, survival curves were constructed, and statistical analyses were conducted using GraphPad Prism version 10.5.0 (GraphPad Software, San Diego, CA, USA). Differences between experimental groups were analyzed using Student’s *t*-test, with *p* < 0.05 considered statistically significant.

### 2.13. Tumor Histopathological Analysis

#### 2.13.1. Tumor Collection

Both control and CAP-treated tumors in the incomplete or non-resection model were collected for analysis 24 h post-CAP treatment.

#### 2.13.2. Tissue Processing and Sectioning

Excised tumor tissues were fixed in 10% neutral buffered formalin for 24 h. Fixed tissues were then dehydrated, embedded in paraffin, and sectioned into 5-μm-thick slices using a microtome.

#### 2.13.3. Bright Field Microscopy

Tumor tissue sections were dewaxed and rehydrated before being stained with hematoxylin and eosin (H&E). Bright-field images were then captured using a light microscope equipped with a digital camera.

#### 2.13.4. Immunohistochemistry (IHC) for Cleaved Caspase-3

Deparaffinized tumor sections were subjected to antigen retrieval using a heat-induced method. After blocking with 5% bovine serum albumin (BSA) (Catalog No. A2153, Sigma-Aldrich, St. Louis, MO, USA), sections were incubated overnight at 4 °C with a primary antibody against cleaved caspase 3 (Catalog No. 9661, Cell Signaling Technology, Danvers, MA, USA). Following washing, sections were incubated with an appropriate secondary antibody conjugated to a fluorophore. Nuclei were counterstained with 4′,6-diamidino-2-phenylindole (DAPI) (Catalog No. D9542, Sigma-Aldrich, St. Louis, MO, USA) according to standard protocol. Immunofluorescent images were captured using a fluorescence microscope.

### 2.14. Statistical Analysis

In the in vitro experiments, comparisons between the control and CAP-treated groups were done using Student’s *t*-test, with statistical significance set at *p* < 0.05 (* *p* < 0.05; ** *p* < 0.01; and *** *p* < 0.001). Results were presented as mean ± standard deviation (SD). Error bars in the graphs represent SD. For the in vivo experiments, survival analysis was performed using the Kaplan–Meier test, and differences in survival between groups were evaluated using the Log-rank (Mantel–Cox) test. A *p* value < 0.05 was considered statistically significant, with *p* values calculated directly from the Log-rank test using GraphPad Prism version 10.5.0 (GraphPad Software, San Diego, CA, USA). Histological quantification graphs were analyzed using Student’s *t*-test, and results are shown as mean ± SD, with *p* < 0.05 considered statistically significant. All experimental procedures were performed following the respective manufacturer’s standard protocols.

## 3. Results

### 3.1. Cold Atmospheric Plasma (CAP) Reduces Proliferation and Viability of SK-N-AS and LAN-5 Cells

**Figure 3:**
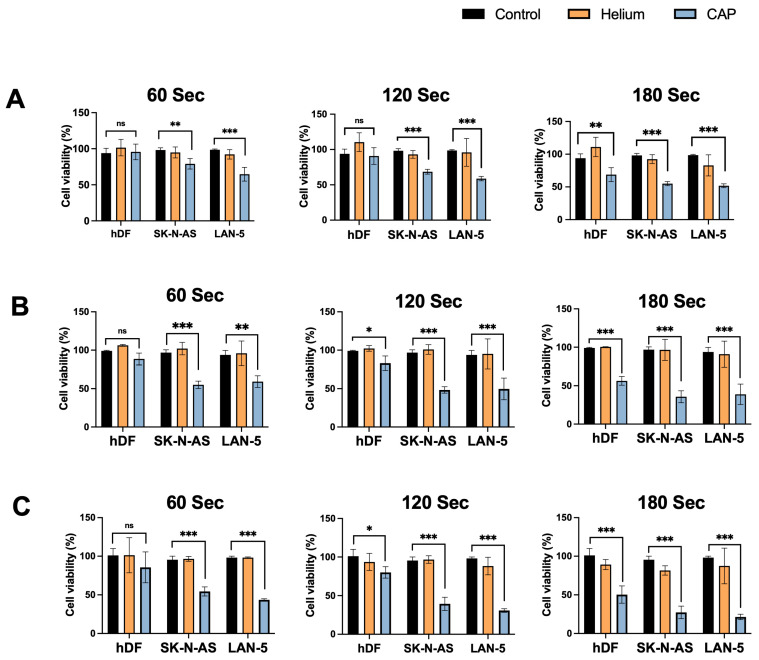
Cold Atmospheric Plasma (CAP) reduces cell viability in NB cells while sparing normal fibroblasts hDF. (**A**–**C**) Relative cell viability (%) of hDF, SK-N-AS, and LAN-5 cells following CAP treatment for 60, 120, and 180 s. Viability was assessed using the CCK-8 assay at 24 h, (**B**) 48 h and (**C**) 72 h post-treatment. Untreated controls and helium-only treated groups were included for comparison. CAP significantly reduced cell viability in NB cells in a dose- and time-dependent manner, with minimal effects on hDFs. Data are presented as mean ± standard deviation (*n* = 3). Statistical significance was determined by Student’s *t*-test (* *p* < 0.05, ** *p* < 0.01, *** *p* < 0.001; ns: not significant).

The effects of CAP on cell viability were assessed using the CCK-8 assay at three separate time points: 24, 48, and 72 h post-treatment. As shown in Figure 3A–C, hDF, SK-N-AS, and LAN-5 cells were treated with CAP for 60, 120, and 180 s, and compared to untreated (control) and helium-only treated cells. At 24 h post-treatment (Figure 3A), SK-N-AS and LAN-5 cells showed reduced viability. The effect was more pronounced at 120 and 180 s, indicating a dose-dependent response. In contrast, hDF cells showed only modest reductions in viability compared with NB cells. This trend was even more pronounced at 48 h (Figure 3B) and 72 h after treatment (Figure 3C). The reductions were statistically significant (* *p* < 0.05, ** *p* < 0.01, *** *p* < 0.001) for both SK-N-AS and LAN-5 cells at all treatment durations beyond 60 s.

Notably, hDF continued to maintain relatively higher viability across all durations. To further evaluate CAP-induced morphological changes, fluorescence microscopy was performed on RFP-labeled hDF and GFP-labeled SK-N-AS cells, as shown in Supplementary Figure S1. Untreated control cells exhibited normal spread morphology and intact adherence. Following 60 s of CAP treatment, SK-N-AS cells began to show early signs of rounding and contraction, while hDFs remained largely unaffected. After 120 s, SK-N-AS cells exhibited more pronounced morphological changes, characterized by a reduction in cell density and increased detachment. By 180 s, SK-N-AS cells appeared highly contracted, spherical, and sparsely distributed—hallmarks of apoptotic or necrotic cell death. Meanwhile, hDF cells showed relatively preserved structure and confluency, indicating minimal susceptibility to CAP under identical conditions.

The analyses demonstrate that CAP selectively reduces the viability of neuroblastoma cells in a dose- and time-dependent manner while sparing normal fibroblasts.

### 3.2. CAP Induces Intracellular ROS

**Figure 4:**
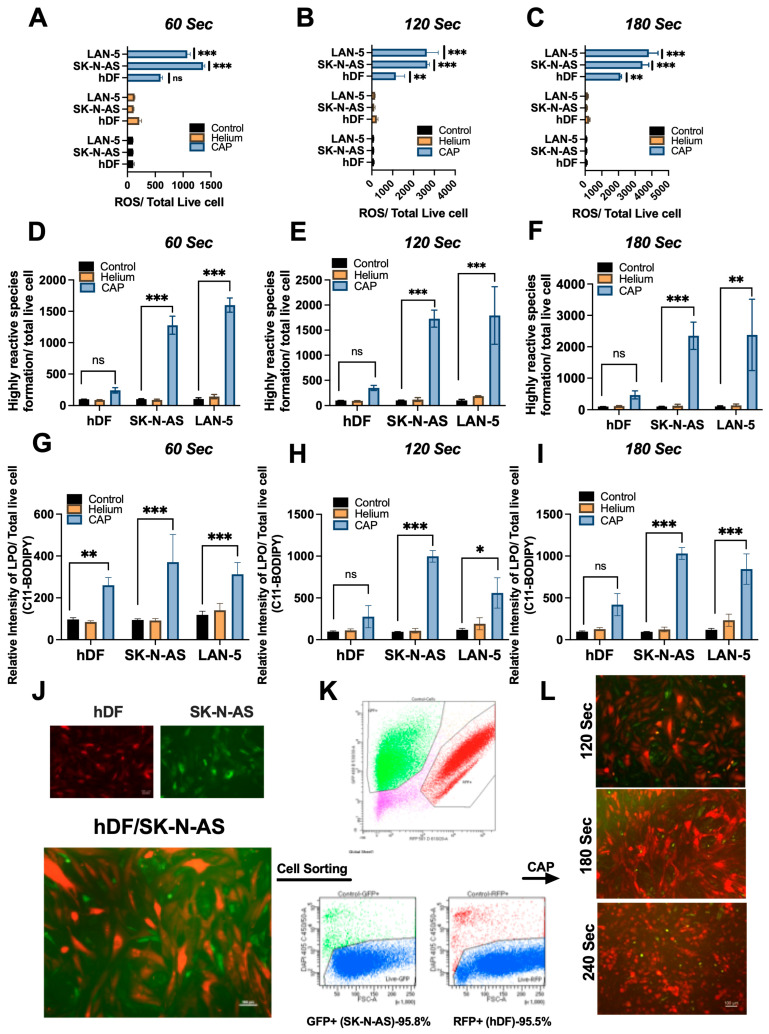
CAP induces intracellular ROS, hydroxyl radicals/peroxynitrite, and lipid peroxidation, and selectively targets NB cells in co-culture. (**A**–**C**) Intracellular ROS levels were quantified using H2DCFDA in hDF, SK-N-AS, and LAN-5 cells treated with CAP for 60, 120, and 180 s. (**D**–**F**) Hydroxyl radicals and/or peroxynitrite were assessed using hydroxyphenyl fluorescein (HPF) under the same treatment durations. (**G**–**I**) Lipid peroxidation was evaluated using the C11-BODIPY 581/591 fluorescence assay, which showed a dose-dependent increase in oxidation, particularly in SK-N-AS and LAN-5 cells. (**J**) Representative images of the co-culture model (Scale bar: 100µm), where GFP-labeled SK-N-AS cells were plated on ~90% confluent RFP-labeled hDFs. (**K**) Flow cytometry-based sorting confirmed that the co-culture contained ~95.8% GFP-positive NB cells and ~95.5% RFP-positive fibroblasts. (**L**) Fluorescence microscopy images (Scale bar: 100µm) post-CAP treatment (120, 180, and 240 s) revealed selective killing of NB cells while fibroblasts remained largely unaffected. Data are presented as mean ± SD and statistical significance was determined by Student’s *t*-test (* *p* < 0.05, ** *p* < 0.01, *** *p* < 0.001; ns: not significant). Modest fibroblast ROS/LPO increases reflect baseline susceptibility but did not result in comparable apoptosis, supporting CAP selectivity.

SK-N-AS and LAN-5 cancer cells exposed to CAP for 60, 120 and 180 s showed significantly heightened intracellular ROS accumulation compared to normal hDF cells under the same treatment (Figure 4A–C). Moreover, upon treatment with CAP for 60, 120 and 180 s, SK-N-AS and LAN-5 cells exhibited a substantial increase in fluorescence emitted by ROS-induced oxidation of DCFH-DA, compared to untreated cells.

### 3.3. CAP Elevates Highly Reactive ROS (hROS) Formation

To identify hROS, such as ^·^OH and ONOO^−^, hDF and NB cells were CAP treated for 60, 120 and 180 s and incubated with HPF (Hydroxyphenyl fluorescein) at room temperature for an hour. HPF itself is not highly fluorescent and highly resistant to light-induced oxidation, but when it reacts with hROS, HPF exhibits strong fluorescence. Our results showed that CAP exposure increased the fluorescence intensity of HPF-loaded SK-N-AS and LAN-5 cells dose-dependently (** *p* < 0.01 and *** *p* < 0.001). Our results suggested that CAP generated hROS formation intracellularly in NB cells and comparatively low fluorescence intensity in hDF cells, as illustrated in Figure 4D–F.

### 3.4. CAP-Induced Lipid Peroxidation Detected by C11-BODIPY Fluorescent Imaging

A significantly and consistently stronger emission pattern of the oxidized green fluorescence was observed with 180 s CAP-treated cancer cells SK-N-AS and LAN-5 (*** *p* < 0.001) when compared to untreated control, helium-treated, and normal cells (hDF) (Figure 4G–I). This trend persisted for 120 s CAP-treated cells, showing a gradual decline in emission intensity with 60 s CAP treatment. These findings collectively confirm a significant increase in oxidized lipids using C11-BODIPY following CAP treatment. The observed changes reflect membrane-level oxidative damage induced by CAP, visualized in live cells using fluorescent microscopy.

### 3.5. Co-Culture Model Demonstrates Selective Cytotoxicity of CAP in NB Cells

To further evaluate the selective cytotoxic effects of CAP treatment, a co-culture model comprising GFP-labeled NB cells SK-N-AS and RFP-labeled hDF fibroblasts was established. Following 24 h co-culture on regular culture plates (Figure 4J), fluorescence imaging confirmed approximately equal cell distribution and viability between the two populations. Subsequent cell sorting analysis validated the co-culture composition, revealing ~95.8% GFP+ neuroblastoma cells and ~95.5% RFP+ fibroblasts (Figure 4K), indicating a balanced and viable system for evaluating CAP-induced responses.

Co-cultures were exposed to CAP for 120, 180, and 240 s. At 48 h post-treatment, the fluorescence images were captured (Figure 4L). Time-dependent cytotoxicity was evident, with a pronounced reduction in GFP+ SK-N-AS viability while RFP+ hDF remained largely unaffected. This differential sensitivity suggests a selective vulnerability of cancer cells to CAP-induced treatment within the co-culture environment.

These findings demonstrate the potential of CAP to selectively target NB cells without adversely impacting adjacent normal cells, highlighting its promise as a localized and tumor-specific therapeutic strategy.

### 3.6. CAP Induces Lipid Peroxidation and Oxidative DNA Damage in NB Cells

**Figure 5:**
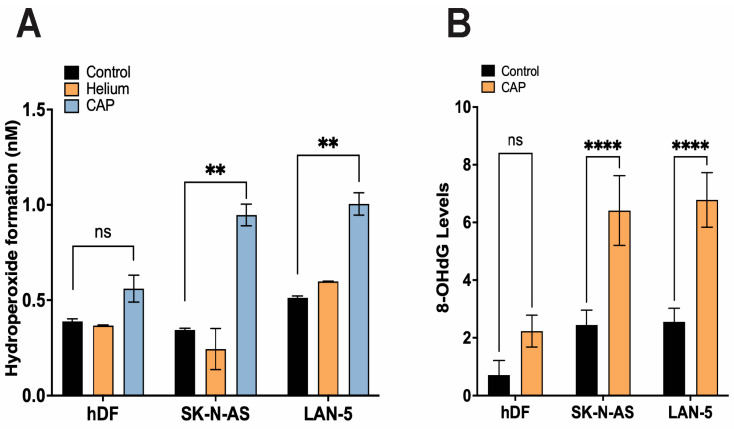
CAP-induced oxidative stress markers in NB cells and fibroblasts. (**A**) Lipid hydroperoxide levels were measured at 48 h post-CAP treatment in hDF, SK-N-AS, and LAN-5 cells. CAP significantly increased lipid hydroperoxide formation in NB cells (*p* < 0.01), with minimal effect on fibroblasts. Dose-dependent increases observed, though baseline variability limited curve separation. (**B**) 8-OHdG, a marker of oxidative DNA damage, was measured in NB cells and fibroblasts after CAP treatment and control, showing a significant increase in CAP-treated SK-N-AS and LAN-5 cells compared to the controls, while hDFs showed no significant change. Significance markers: ** *p* < 0.01, **** *p* < 0.0001. Data are presented as mean ± SD and statistical significance was determined by Student’s *t*-test.

Lipid hydroperoxide levels were significantly elevated in CAP-treated SK-N-AS and LAN-5 NB cells, indicating increased membrane lipid peroxidation and oxidative stress following treatment (Figure 5A). This biochemical assessment demonstrated a dose-dependent accumulation of peroxidized lipids in NB cells, while levels remained comparatively low in normal hDFs. Additionally, 8-hydroxy-2′-deoxyguanosine (8-OHdG) quantification revealed a marked increase in oxidative DNA lesions in CAP-treated NB cells, confirming that CAP not only affects cellular membranes but also induces nuclear DNA damage (Figure 5B). These findings support CAP’s ability to trigger oxidative stress at multiple cellular levels in a cancer-selective manner.

### 3.7. CAP Elicits Apoptosis in NB Cells

**Figure 6:**
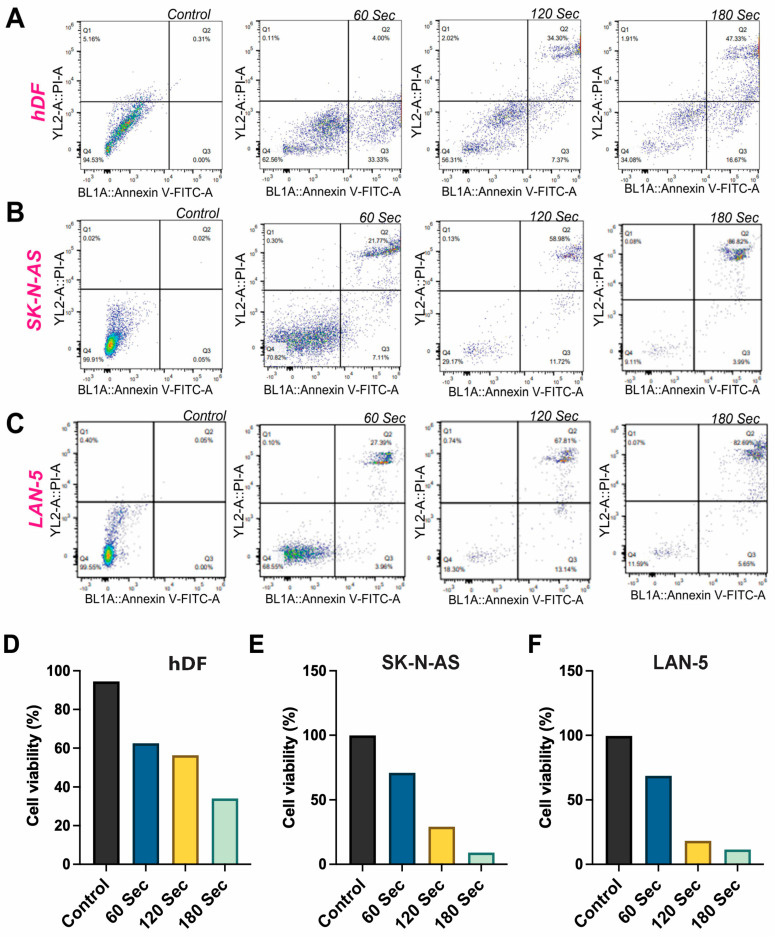
CAP induces apoptosis and reduces cell viability in NB cells. (**A**–**C**) Annexin V/PI flow cytometry plots of hDF (**A**), SK-N-AS (**B**), and LAN-5 (**C**) cells following CAP treatment for 0, 60, 120, and 180 s. Apoptotic populations increased with treatment duration in NB cells, while hDFs showed a limited response in the Annexin V/PI plots (**A**–**C**), lower left quadrants (Annexin V^−^/PI^−^) represent viable cells, lower right quadrants (Annexin V^+^/PI^−^) represent early apoptotic cells, upper right quadrants (Annexin V^+^/PI^+^) represent late apoptotic or necrotic cells, and upper left quadrants (Annexin V^−^/PI^+^) represent necrotic cells. (**D**–**F**) Quantification of cell viability in hDF (**D**), SK-N-AS (**E**), and LAN-5 (**F**) cells confirms a dose-dependent reduction in viability in NB cells, with minimal effect on normal fibroblasts. Data shown represents single representative results. BL1A and V-FITC-A correspond to the FITC (Annexin V) fluorescence channel (excitation 488 nm, emission 530 nm), and YL2-A/PI-A correspond to the propidium iodide (PI) fluorescence channel (excitation 561 nm, emission 610 nm). These labels represent the flow-cytometer detector channels used to distinguish Annexin V-FITC-positive and PI-positive cell populations.

Figure 6A–F showed significant contrast in the response of normal hDF and NB cancer cells (SK-N-AS and LAN-5) to CAP treatment. Initially, in the control groups, both hDF and NB cells exhibit robust viability, with hDF displaying 94.53% and NB cells showing over 99.5% healthy cells, accompanied by minimal apoptotic activity (hDF: 0.31%, SK-N-AS: 0.02%, LAN-5: 0.06%). However, after CAP treatment for 60, 120, and 180 s, distinct differences emerged. Throughout the treatment, hDF cells consistently maintain a higher proportion of healthy cells compared to NB cells. For instance, after 180 s of CAP treatment, hDF cells retain 34.08% healthy cells, while SK-N-AS and LAN-5 cells show a substantial decrease to 9.11% and 11.59% healthy cells, respectively. Conversely, apoptotic rates escalate more significantly in cancer cells across all time durations. Following 180 s of CAP treatment, hDF cells exhibit an apoptotic rate of 64%, significantly lower than SK-N-AS (90.81%) and LAN-5 (93.94%) cells. Data shown represents single representative results; however, the assays were independently replicated, and quantitative values were analyzed as mean ± SD. Statistical analysis consistently demonstrated a highly significant increase in apoptosis in neuroblastoma cells compared with hDFs (*p* < 0.001), though replicate data are not displayed. The data suggest CAP treatment’s preferential targeting of NB cells, resulting in a notable decrease in viability and a marked increase in apoptotic activity. In contrast, hDF cells display a more resilient response, maintaining higher viability and lower apoptotic rates even under prolonged CAP exposure.

### 3.8. CAP-Induced Modulation of Cell Cycle Progression

**Figure 7:**
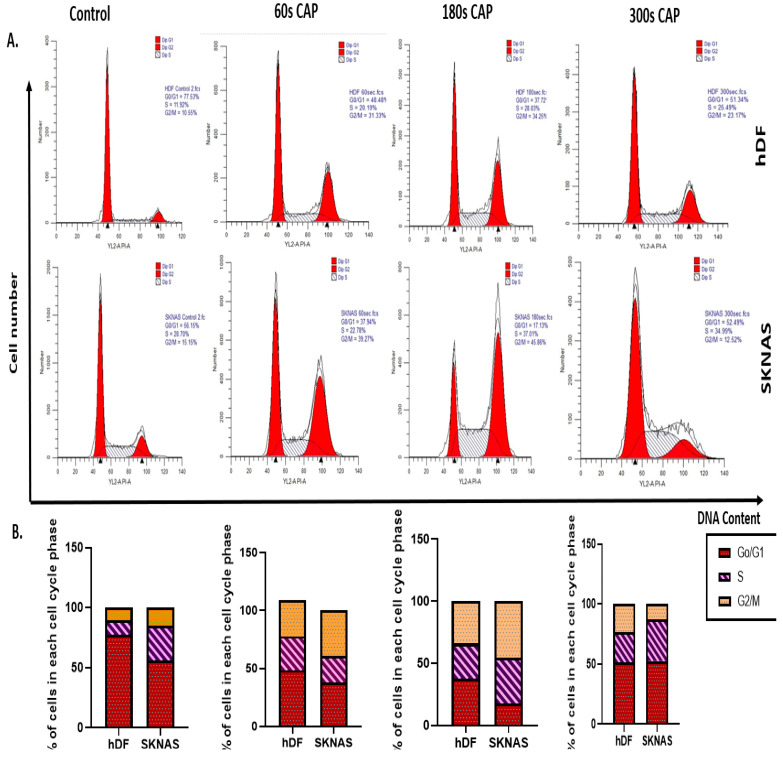
CAP alters cell cycle distribution in fibroblasts and NB cells. (**A**) Representative histograms from flow cytometry analysis showing DNA content profiles of hDF and SK-N-AS cells under control conditions and after CAP treatment for 60, 180, and 300 s. Cell populations in G0/G1, S, and G2/M phases were distinguished. (**B**) Quantitative bar graphs representing the percentage of cells in each cell cycle phase for both cell types across all treatment durations. CAP induces notable G2/M arrest in SK-N-AS cells and modulates phase distribution in hDF cells. At 300 s, SK-N-AS cells showed partial redistribution, likely due to checkpoint adaptation, contrasting with a stronger arrest at 180 s. Modest fibroblast ROS/LPO increases reflect baseline susceptibility but did not result in comparable apoptosis, supporting CAP selectivity. The data shown represent single representative results for the present study.

The cell cycle analysis post-CAP treatment revealed differential effects between normal hDF and NB cells (SK-N-AS) across various exposure durations (60, 180, and 300 s), as shown in Figure 7A,B. In hDF cells, CAP exposure resulted in a notable reduction in the G0/G1 phase (from 77.55% in control to 48.49% at 60 s)**,** accompanied by corresponding increases in the S (from 11.92% to 20.19%) and G2/M (from 10.55% to 31.33%) phases. These alterations persisted at 180 s (G0/G1: 37.72%, S: 28.65%, G2/M: 34.25%) and 300 s (G0/G1: 51.34%, S: 25.49%, G2/M: 23.17%), with hDF maintaining a relatively balanced distribution across phases. In SK-N-AS cells, CAP led to a pronounced accumulation in the G2/M phase (increasing from 15.15% in control to 39.27% at 60 s and 45.86% at 180 s), along with a consistent reduction in G0/G1 populations (from 56.15% in control to 11.73% at 180 s). By 300 s, partial redistribution occurred, with SK-N-AS cells showing recovery of G0/G1 (52.49%) but still elevated S phase (34.99%) compared to control. These data indicate that CAP disrupts cell cycle distribution in a time-dependent manner, particularly enhancing G2/M arrest in NB cells, which may contribute to its anti-proliferative effect.

### 3.9. In Vivo CAP Treatment and Its Response

#### 3.9.1. CAP Treatment Following Incomplete Surgical Resection

**Figure 8:**
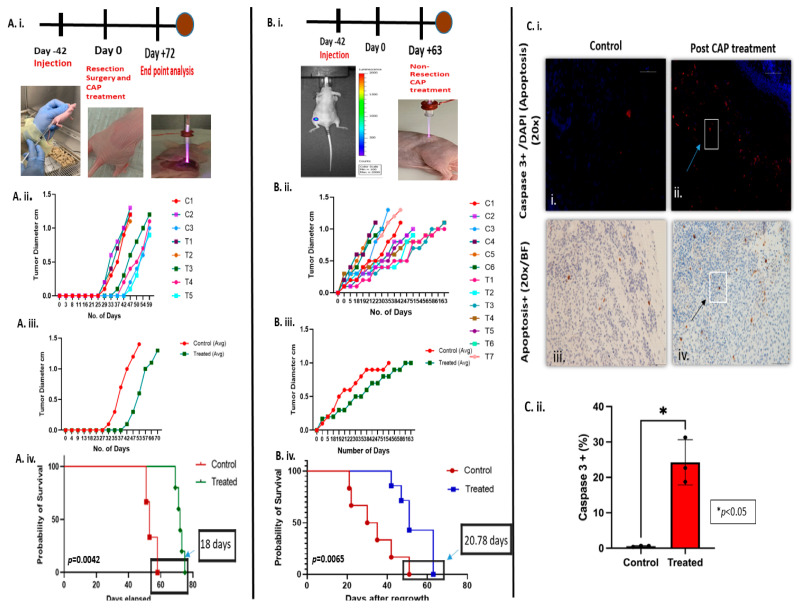
In vivo effects of CAP treatment on tumor regrowth, survival, and apoptosis in in vivo NB models. (**A. i**) In the resection model, mice were injected with tumor cells SK-N-AS on the flank. After the tumor reached 1–3 mm in size, mice underwent incomplete surgical resection followed by CAP treatment and were analyzed at the endpoint. (**A. ii**,**iii**) Tumor growth curves from individual animals and average diameter show reduced tumor regrowth in CAP-treated mice (T1–T5) compared to controls (C1–C3). (**A. iv**) Kaplan–Meier survival analysis indicated a significant extension in survival in the CAP-treated group compared to controls (*p* = 0.0042), corresponding to an increase in median survival of 18 days. (**B. i**) In the non-resection model, mice received tumor cell injections, and the tumor was monitored. When the tumor reached 1–3 mm, they were treated with CAP (no resection). When the tumors reached 2 cm or the mice were moribund, they were euthanized, and the tumors were collected and analyzed. (**B. ii**,**iii**) Tumor diameter progression in individual animals (control: C1–C6; treated: T1–T7) and average tumor diameter showed markedly slower growth in the CAP-treated group. (**B. iv**) Survival analysis revealed a significant extension of post-regrowth survival in treated mice (*p* = 0.0065), corresponding to an increase in median survival of 20.78 days. (**C**) Histological evaluation of tumors post-CAP treatment revealed enhanced apoptosis in treated tissues. (**C. i**(i,ii)) Immunofluorescence staining images (Scale bar: 50 µm) showed increased expression of cleaved caspase-3 (red) in CAP-treated tumors compared to minimal expression in controls, with nuclei counterstained using DAPI (blue). (**C. i**(iii,iv)) Bright-field immunohistochemistry images (Scale bar: 50 µm) confirmed greater apoptotic activity in CAP-treated tumors versus controls. (**C. ii**) Quantification of cleaved caspase-3-positive cells demonstrated a significant increase in apoptotic cells in the CAP-treated group compared to controls (*p* < 0.05).

In the resection study, mice received CAP treatment immediately after surgical resection of approximately 95% of the tumor volume (Figure 8A(i)). The schematic and representative images illustrate the surgical and CAP-treatment procedure. Tumor diameter measurements (Figure 8A(ii)–(iii)) revealed a slower regrowth trajectory in CAP-treated mice compared to untreated controls. While all animals eventually exhibited regrowth, the rate of regrowth was significantly delayed in the CAP-treated group. Survival analysis (Figure 8A(iv)) indicated a significant increase in overall survival for CAP-treated mice (*p* = 0.0042), demonstrating that CAP application at the residual tumor following resection can delay tumor regrowth and extend survival.

#### 3.9.2. CAP Treatment Following Non-Surgical Resection

In the non-resection cohort, CAP was applied directly to established tumors without surgical intervention (Figure 8B(i)), which can be considered as primary treatment. Tumor growth and maximal diameter (Figure 8B(ii,iii)) were reduced in the CAP-treated group compared to controls. Survival analysis (Figure 8B(iv)) again revealed increased survival in the CAP-treated group (*p* = 0.0065). This data suggest that CAP can be considered a standalone treatment modality in controlling tumor progression, even in the absence of resection.

#### 3.9.3. Histopathological Analysis Post-CAP Treatment

Histopathological evaluation was performed to assess cellular responses to CAP treatment in non-resection tumor models. Immunohistochemistry for cleaved caspase-3, a marker of apoptosis, revealed a substantial increase in apoptotic cells in the treated tumors compared to controls. In the study, immunofluorescent staining 24 h post-CAP treatment (Figure 8C(i)(i,ii)) showed markedly higher caspase-3 expression (red) in treated tumors, co-localizing with nuclear DAPI staining (blue), indicating increased apoptotic activity. Similarly, apoptotic staining in a bright field (Figure 8C(i)(iii,iv)) corroborated these findings, showing prominent brown-stained apoptotic cells in treated sections. Quantitative analysis (Figure 8C(ii)) confirmed a significant increase in the percentage of caspase-3^+^ cells in the treated group compared to the controls, suggesting the effective induction of apoptosis by CAP. Together, these histopathological findings substantiate the pro-apoptotic effect of CAP and support its potential in controlling tumor recurrence by directly inducing cell death. Additionally, CAP-mediated cytotoxicity may involve downstream signaling pathways such as the p53/p73 axis, PI3K/AKT, and MAPKs, as well as modulation of neurotrophin receptors (TrkA/B and p75NTR), which are known to regulate oxidative stress responses in NB. Future approaches could explore CAP in combination with pro-oxidant agents or inhibitors of antioxidant systems such as glutathione metabolism, to enhance tumor-selective responses and broaden translational potential.

## 4. Discussion

NB is a challenging solid tumor that afflicts many children. For high-risk tumors, the treatment is extensive and involves many modalities such as neoadjuvant chemotherapy, surgery, bone marrow transplant, radiation therapy, and immunotherapy. Surgery remains one of the critical modalities for the successful outcome of patients with high-risk NB, as more extensive resection, which is often considered more than 90% resection of the original tumor volume, has been shown to improve long-term event-free survival [17]. However, these tumors often encase vital structures such as major blood vessels and nerve roots, making more complete resection dangerous and often associated with increased complications such as bleeding, unintended nephrectomy, and chyle leak.

In this research, we are exploring CAP as another potential modality in the treatment of high-risk NB. Since CAP has to be applied directly to the tumor, it would ideally be applied only when the tumor is under direct visualization, which can occur during surgical biopsy or resection. Previous studies [13] have demonstrated that CAP is effective against murine neuroblastoma (Neuro2a) in syngeneic, immunocompetent A/J mice, establishing its antitumor efficacy in an intact immune environment. Building upon that foundational work, the current study was designed to focus on human NB cell lines (SK-N-AS and LAN-5 in in vitro experiments and SK-N-AS in in vivo xenograft models). The primary objective of this study was to elucidate CAP’s mechanistic and selective effects on human NB cells as a critical translational step toward clinical application. In our present research, we investigated the ability of CAP to selectively target tumor cells over normal cells. In the incomplete resection model, we resected about 90% of the tumor to simulate the 90% surgical resection typically observed in a clinical setting. We also tested CAP’s ability to target tumors without resection.

The results of these experiments are very promising. The observed reductions in proliferation and viability of NB cells following CAP treatment align with previous studies demonstrating the cytotoxic effects of CAP on the two NB cell lines that we chose, SK-N-AS and LAN-5 [12,14]. We attempted to explore the underlying mechanisms responsible for these effects. There are many possibilities. However, given the nature of CAP, which produces RONS, the increase in intracellular ROS accumulation and lipid peroxidation indicates oxidative stress-induced damage, consistent with the mechanism underlying CAP-induced apoptosis [10,16]. The significant elevation in lipid hydroperoxide levels post-CAP treatment further underscores the oxidative stress burden imposed on cancer cells, potentially contributing to cell death [18]. Additionally, these effects further implicate oxidative stress pathways in NB [16]. Furthermore, the induction of DNA damage, as evidenced by elevated levels of 8-OHdG, highlights the genotoxic effects of CAP treatment on NB cells [19,20,21]. Future work will include ROS rescue experiments using scavengers such as N-acetylcysteine (NAC) and complementary pro-oxidant challenges, to directly confirm the causal role of ROS in CAP-induced apoptosis.

The differential responses observed between NB cells and normal hDF cells, particularly in terms of viability, apoptotic rates, and alterations in cell cycle dynamics, underscore the selective cytotoxicity of CAP towards NB cells [22]. The ability of CAP to induce apoptosis specifically in NB cells while sparing normal hDF cells holds considerable therapeutic potential, offering a promising approach for the development of targeted cancer therapies with minimal off-target effects, as demonstrated in other cancer cells [23]. Using Annexin V/PI data, we clarified that apoptosis is a programmed form of cell death distinct from necrosis, which is caused by uncontrolled external damage. Our findings support apoptosis as the primary mode of CAP-induced cell death, with secondary necrosis occurring only at extended exposures. A distinct pre-G1 peak was not detected, consistent with NB, where apoptosis is more reliably assessed by Annexin V/PI rather than pre-G1 fractions. Future work will also delineate intrinsic apoptotic markers (Bax/Bcl-2, Caspase-9) and extrinsic markers (FADD/Fas, Caspase-8) to clarify the upstream signaling cascades engaged by CAP. Furthermore, the ability to modulate cell cycle dynamics in NB cells suggests that CAP treatment could be tailored to disrupt NB cell proliferation, providing a rationale for further investigation into the mechanistic underpinnings of CAP-induced cell cycle alterations and their therapeutic implications [24]. Notably, the cell cycle analysis assay utilized a slightly different set of CAP exposure times, which were chosen based on preliminary optimization studies to enhance detection of cell cycle phase-specific effects. Similarly, in co-culture experiments, treatment durations of 120, 180, and 240 s were selected to assess selective cytotoxicity under more physiologically relevant and prolonged exposure conditions, enabling clearer differentiation between tumor and normal cell responses. Importantly, our co-culture experiments further confirmed the selectivity of CAP, demonstrating that CAP effectively induced apoptosis in GFP-labeled NB cells while sparing RFP-labeled normal fibroblasts within the same microenvironment. This reinforces CAP’s potential to target tumor cells specifically without damaging surrounding healthy tissues, a crucial aspect for future localized therapies. The future studies will include additional assays across multiple post-incubation hours to further strengthen mechanistic insights.

Our incomplete resection model highlights the benefit of combining CAP with surgical tumor resection to enhance local disease control in NB. Local recurrence in NB remains a major therapeutic hurdle, often leading to severe clinical complications such as pain and organ dysfunction. While recurrence was observed in all animals, CAP-treated groups exhibited delayed tumor regrowth and increased caspase-3-mediated apoptosis at the tumor site, with minimal damage to surrounding tissues. This supports CAP’s role as an adjunctive therapy that enhances surgical outcomes by targeting residual disease [15,22]. These in vivo results highlight that although CAP alone does not prevent recurrence, it can reduce the rate of tumor regrowth when applied locally post-resection, thus emphasizing CAP as a supplement to, rather than a replacement for, surgical resection. This principle suggests that CAP’s most realistic and immediate clinical application is adjunct therapy at the time of surgical resection. These findings align with the overarching goal of improving local disease management, particularly in high-risk NB, where local recurrence contributes to significant morbidity [17]. CAP’s local application may offer symptom relief and improved control of residual tumor burden when integrated into a multimodal treatment framework, especially when located near critical vascular or neural structures, where complete surgical resection poses significant risks of morbidity. The selective cytotoxicity of CAP toward tumor cells, while sparing normal tissues, positions it as a potential adjunct intraoperative tool for precision therapy. Further preclinical validation is warranted to assess CAP’s safety profile on normal endothelial and neuronal cells before clinical translation in these challenging surgical contexts.

While CAP’s mechanisms are described in terms of oxidative stress and apoptosis, the role of the tumor microenvironment and the immune response in modulating CAP’s effects remains underexplored. Future work should consider how CAP interacts with the immune landscape and extracellular matrix, as this may influence treatment outcomes, particularly in orthotopic or immunocompetent models [25,26,27]. Recent studies further underscore this point, which highlights how plasma-derived oxidants can trigger immune-modulatory pathways that enhance antitumor responses [28] along with a comprehensive framework for understanding plasma-induced redox chemistry in the biomedical context [16]. These works support our conclusion that CAP’s effects are not limited to direct cytotoxicity but also extend to shaping the tumor microenvironment and systemic responses, emphasizing the translational importance of integrating CAP into multimodal oncology strategies.

Future studies should employ syngeneic, humanized, immunocompetent mouse models or more complex human organoid models to evaluate CAP’s immunomodulatory potential responses to establish human-relevant tumor responses as a foundation for subsequent immunologic studies. Additional research and clinical trials are needed to better understand the mechanisms of CAP therapy, optimize treatment regimens, and evaluate long-term outcomes in NB and other solid tumors. These investigations underscore the potential of CAP as a valuable addition to existing treatment strategies, particularly for high-risk patients requiring enhanced local control [7,17], and highlight its promise as a novel therapeutic approach for this aggressive pediatric malignancy.

### Challenges and Limitations

This study is limited by the use of established NB cell lines and fibroblasts, which do not fully replicate the complexity of the in vivo tumor microenvironment. Accordingly, the findings should be interpreted as a mechanistic proof-of-concept. Viability differences in fibroblasts were described as a modest reduction rather than unaffected, in line with the data. In addition, the use of subcutaneous, immunodeficient tumor models does not fully capture the biological and immune complexity of NB in its native context. While CAP demonstrated selective cytotoxicity and induction of apoptosis, the long-term impact on normal tissues and immune responses was not assessed. CAP dosing was standardized across models, but further optimization with additional post-treatment times will be required for clinical translation. We acknowledge the small xenograft cohort size and stress the need for orthotopic and immunocompetent NB models, as well as extended mechanistic analyses in future studies, to strengthen the translational relevance of these findings. Future in vivo studies will employ syngeneic, immunocompetent models to evaluate immune responses alongside tumor control. CAP penetration depth is limited, highlighting its role in localized applications and the importance of optimizing parameters to minimize potential off-target effects on normal tissue.

## 5. Conclusions

CAP treatment exhibited significant effects on NB cancer cells both in vitro and in vivo. We demonstrated a significant reduction in proliferation and viability, increased intracellular ROS accumulation, augmented lipid peroxidation, drastic increases in lipid hydroperoxide levels, induction of DNA damage, initiation of apoptosis, and alterations in cell cycle dynamics in in vitro experiments. Additionally, the co-culture model strongly supported CAP’s selective cytotoxic action, reinforcing its precision in targeting cancer cells while sparing surrounding normal tissues.

We also showed slower tumor regrowth after incomplete resection with CAP treatment and slower tumor growth after non-resection with CAP treatment in vivo models. These results further highlight that while CAP alone does not prevent recurrence or be used as primary therapy, its localized application post-resection delays regrowth and promotes apoptosis at the tumor margin, suggesting it may serve as a valuable adjunct to surgical strategies in managing residual disease, especially near critical vascular structures where surgical resection is challenging and reducing the risk of symptomatic local relapse. Together, these findings support the use of CAP as a complementary therapeutic strategy to surgery, enhancing local control of NB with minimal off-target effects.

## Data Availability

The raw data supporting the conclusions of this article will be made available by the authors on request.

## Supplementary Materials

The following supporting information can be downloaded at https://www.mdpi.com/article/10.3390/cancers17213432/s1. Figure S1: Representative fluorescence microscopy images of RFP-labeled hDFs (top row) and GFP-labeled SK-N-AS cells (bottom row) following CAP treatment for 60, 120, and 180 s. Images were captured 48 h post-treatment. CAP exposure induced progressive morphological alterations and detachment in SK-N-AS cells, while Fibroblasts maintained spread morphology and adherence, without pronounced contraction or detachment. Representative cell images are shown to illustrate qualitative context and morphological differences between treated and control groups.
