# Supplementary material for: Cold Atmospheric Plasma Selectively Targets Neuroblastoma: Mechanistic Insights and In Vivo Validation
Source: Cancers (Basel). 2025 Oct 25;17(21):3432. doi: 10.3390/cancers17213432 (PMC12607432; doi:10.3390/cancers17213432)
Supplement: Supplementary file 1 [file cancers-17-03432-s001.zip › cancers-3892065-supplementary.pdf]

cancers
MDPI

Supplementary Data

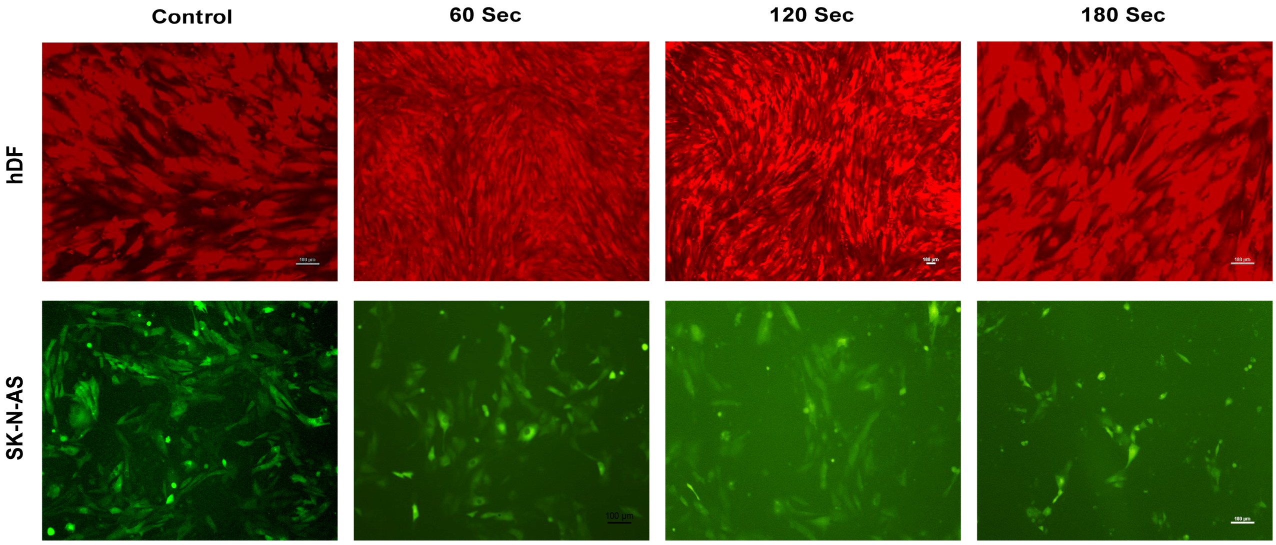

Supplementary Figure S1. Representative fluorescence microscopy images of RFP-labeled hDFs (top row) and GFP-labeled SK-N-AS cells (bottom row) following CAP treatment for 60, 120, and 180 s. Images were captured 48 h post-treatment. CAP exposure induced progressive morphological alterations and detachment in SK-N-AS cells, while Fibroblasts maintained spread morphology and adherence, without pronounced contraction or detachment. Representative cell images are shown to illustrate qualitative context and morphological differences between treated and control groups.
